# Computer-Interpretable Quality Indicators for Intensive Care Medicine: Development and Validation Study

**DOI:** 10.2196/77077

**Published:** 2025-09-26

**Authors:** Falk von Dincklage, Viktor Karl Bublitz, Oliver Kumpf, Carlo Jurth, Reimer Riessen, Maria Deja, Christiane Maria Schewe, Dirk Schädler, Christian Fuchs, Sebastian Gibb, Christian Scheer, Jens-Christian Schewe, Hartmuth Nowak, Felix Balzer, Michael Adamzik, DIVI Information Technology Section, DIVI Quality and Economy Section, Gernot Marx, Gregor Lichtner

**Affiliations:** 1 Department of Anesthesia, Intensive Care, Emergency and Pain Medicine University Medicine Greifswald Greifswald Germany; 2 Department of Anesthesiology and Intensive Care Medicine (CCM/CVK) Charité - Universitätsmedizin Berlin, Corporate Member of Freie Universität Berlin and Humboldt-Universität zu Berlin Berlin Germany; 3 Department of Internal Medicine, Intensive Care Unit University Hospital Tübingen Tübingen Germany; 4 Department of Anesthesiology and Intensive Care Medicine University Hospital Schleswig-Holstein Campus Lübeck Lübeck Germany; 5 Department of Anesthesiology, Intensive Care Medicine and Pain Medicine University Medical Center Rostock Rostock Germany; 6 Department of Anesthesiology and Intensive Care Medicine University Hospital Schleswig-Holstein Campus Kiel Kiel Germany; 7 Center for Artificial Intelligence, Medical Informatics and Data Science University Hospital Knappschaft Kliniken Bochum Bochum Germany; 8 Institute of Medical Informatics Charité - Universitätsmedizin Berlin, Corporate Member of Freie Universität Berlin and Humboldt-Universität zu Berlin Berlin Germany; 9 Department of Anesthesiology, Intensive Care and Pain Medicine University Hospital Knappschaft Kliniken Bochum Bochum Germany; 10 See Acknowledgments; 11 Department of Intensive Care Medicine and Intermediate Care University Hospital RWTH Aachen Aachen Germany

**Keywords:** intensive care medicine, quality indicator, clinical quality assessment, electronic quality measures, FHIR, Fast Healthcare Interoperability Resources, semantic interoperability, computer-interpretable rules, standardization

## Abstract

**Background:**

Quality indicators (QIs) can help assess intensive care quality, identify potential for improvement, and ultimately enhance patient outcomes. Therefore, the German Interdisciplinary Association of Critical Care and Emergency Medicine (DIVI) has developed QIs for intensive care medicine. However, variability in how these are technically implemented across health care facilities currently limits their comparability.

**Objective:**

The aim of the study is to develop unambiguous computer-interpretable representations of the DIVI QIs for intensive care medicine using Fast Healthcare Interoperability Resources (FHIR) and to establish a replicable process for translating narrative QIs into standardized digital formats.

**Methods:**

We first decomposed the narrative DIVI intensive care medicine QIs into two sets of semantic concepts that characterize (1) the targeted patient population and (2) the care aspect specified by each indicator. We mapped the concepts to international vocabularies, defining a supplementary code system for concepts not appropriately represented in existing vocabularies. The decomposed and semantically mapped QIs were then implemented in FHIR using an implementation guide we previously developed to represent clinical practice guideline recommendations. As the translation process holds risks of inducing logical and semantic deviations, the final FHIR representations were back-translated into a narrative form and reviewed with clinical experts, including the authors of the original QIs. The decomposition and semantic mapping were iteratively adjusted based on the experts’ feedback until the results accurately reflected the original intent of the QIs.

**Results:**

The 10 DIVI QIs were decomposed into 31 separately measurable indicators, including 9 structural indicators, 17 process indicators, and 5 outcome indicators. All process and outcome indicators were successfully specified as computer-interpretable representations in FHIR. In total, 58 unique medical concepts were used, of which 52 (90%) could be mapped to concepts from international vocabularies. The remaining 6 concepts—mostly intensive care unit–specific scores or roles—were defined in a supplementary code system. Nested Boolean logic and temporal conditions were fully supported using standard FHIR mechanisms. After iterative adjustments, the final representations were approved as accurate representations of the DIVI QIs by the clinical expert panel.

**Conclusions:**

Our work demonstrates that the structured process developed here enables the unambiguous, computer-interpretable representation of QIs for intensive care. These representations can be used in automated quality management systems to standardize quality assessments across health care facilities. Our newly defined structured process can serve as a blueprint for similar efforts in other specialties. The here-developed computer-interpretable QIs are openly available for reuse and ongoing maintenance. Future work will focus on piloting these indicators in real-world clinical systems and extending the framework to include structural indicators.

## Introduction

Despite vast advances in medical knowledge and technology over the last decades, these advances do not consistently translate into improved patient outcomes and quality of life, as the available treatment options are not always exploited to their full potential in individual patients [1]. The quality of care represents the degree to which these available options are implemented [2]. To systematically improve the quality of medical and nursing care, structured quality management approaches are required. Such approaches include that health care organizations critically reflect on their structural characteristics, processes, and outcomes with a focus on identifying potential for improvement. This can be effectively supported by objective measures that reliably capture the current degree of quality of care: the quality indicators (QIs) [3]. QIs are commonly categorized into structural, process, and outcome indicators. Structural indicators measure the structural requirements for high-quality care, such as facilities, equipment, and staffing. Process indicators measure the quality of the treatment procedures, focusing on whether care aligns with current evidence, guidelines, and standards. Outcome indicators measure treatment results, including the patient’s state of health, quality of life, and satisfaction with the care provided [2].

Since 2010, QIs for intensive care medicine in the German health care system have been developed and published by the German Interdisciplinary Association of Critical Care and Emergency Medicine (DIVI). The current version, revised in 2023, includes 10 indicators that cover structural, process, and outcome-related aspects of intensive care medicine, with each indicator comprising multiple components [4]. Despite their wide application for systematic quality monitoring and improvement processes in intensive care units across Germany, comparisons between different facilities and quality processes across hospitals remain unavailable. A key challenge is that although the QIs are defined in a level of detail comparable to clinical practice guidelines, this still leaves some specifics open to interpretation. As a result, actual implementations of systems to automatically evaluate whether QIs are fulfilled are using slightly different interpretations of the QIs. In consequence of this, benchmark target values for the fulfillment rate of QIs lose their meaning, as even slight differences in the interpretation of a QI can significantly influence the observed fulfillment rates. Furthermore, cross-site quality improvement processes require consistent implementation across the participating institutions to ensure comparability. For example, a quality ranking based on the fulfillment rate of QIs would lose validity in the case of inconsistent implementations. This challenge is not unique to Germany; international efforts have similarly highlighted that inconsistent definitions of QIs hinder benchmarking and comparability across intensive care settings [5,6]. In the United States, for example, the Centers for Medicare & Medicaid Services identified inconsistent specifications of electronic clinical quality measures as a key obstacle to standardized quality assessment [7].

Therefore, precise specifications of QIs, ideally as computer-interpretable rules, are required to facilitate standardized implementations and allow comparisons across health care facilities. Providing a reliable source of such computer-interpretable rules not only ensures uniform implementations across health care facilities but also reduces the effort to update these systems when the QIs are revised [8]. This could be facilitated by publishing QIs not only in a narrative format aligned with text-based clinical guideline recommendations but also in a digital format, formalized at a level of detail comparable with computer-interpretable clinical practice guidelines. In recent years, international initiatives have increasingly adopted shared standards to support such computer-interpretable formats, most notably the Health Level Seven (HL7) Fast Healthcare Interoperability Resources (FHIR) standard. FHIR-based representations of clinical practice guidelines and electronic clinical quality measures are now being used in national and global projects to support quality measurement, benchmarking, and guideline implementation [9,10].

We have recently developed a format for the computer-interpretable representation of clinical practice guidelines that is based on FHIR [11]. Although QIs and clinical practice guideline recommendations are closely related, the format has not been evaluated for its suitability to represent QIs. Furthermore, no structured process currently exists for developing trustable computer-interpretable representations of QIs, that is, representations that are clinically validated through iterative review by multiple domain experts to ensure alignment with the original clinical intent.

Therefore, the primary aim of this study was to develop computer-interpretable representations of the process and outcome QIs for intensive care medicine in Germany based on our FHIR format. As part of the study, we evaluated and extended the format to allow a full representation of QIs. Furthermore, we designed a structured process for developing trustable computer-interpretable representations of QIs by stepwise formalization and repeated integration of clinical experts in the development process.

## Methods

To enhance transparency and reproducibility, this study was reported in accordance with the STARE-HI (Statement on the Reporting of Evaluation Studies in Health Informatics) checklist (Multimedia Appendix 1).

### A Structured Process to Develop Trustable Digital QIs

**Figure 1:**
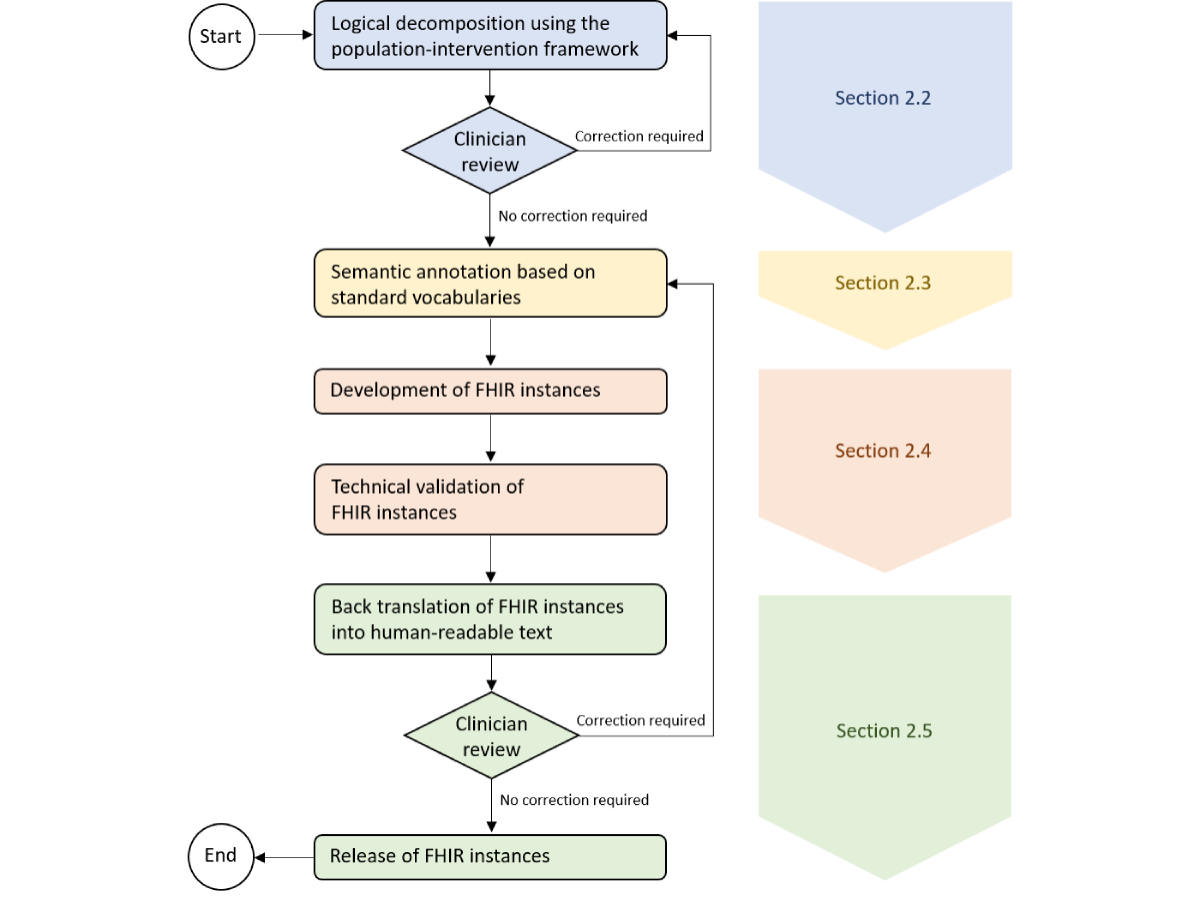
Structured development process for trustable digital representations of quality indicators. FHIR: Fast Healthcare Interoperability Resources.

Developing trustable computer-interpretable representations of QIs requires a close collaboration of medical informatics specialists and clinical experts. While the expertise of medical informatics specialists is required to perform the logical and semantical specification as well as to implement the QIs in structured code, the expertise of clinical experts is required to ensure that the developed representation truly matches the initial intent of the QI. To integrate the expertise of both specialist groups in an effective and efficient process, we separated the development into 4 steps performed by the medical informatics specialists, with 2 reviews performed by the clinical experts to confirm the results, 1 after the first, and 1 after the last step (Figure 1).

In the first step, each QI is logically decomposed using the population-intervention framework (Section 2.2). This is followed by a clinician review to ensure that the result aligns with the original QI. If deviations are identified, the required corrections are communicated back to the team responsible for the logical decomposition, who then repeats the step while incorporating the necessary changes. Once the logical decomposition is accepted, the relevant medical concepts are annotated using standard vocabularies (Section 2.3). Based on this semantic annotation, FHIR instances are created as digital representations of the QIs and then validated (Section 2.4). To ensure that the FHIR-encoded structured representations accurately reflect the intent of the original narrative QIs, the FHIR-encoded representations are back-translated into a format that can be understood and reviewed by clinicians (Section 2.5). If discrepancies are identified during this review, the necessary corrections are applied by revisiting the semantic annotation and FHIR development steps. This iterative process continues until the FHIR representations accurately reflect the narrative definitions. Once finalized, the FHIR instances are released.

### Logical Formalization of the QIs

The content of evidence-based clinical practice guideline recommendations can be decomposed using the PICO (population, intervention, comparison, outcome framework by defining formalized terms for the population, that is, the characterization of the group of patients to whom the recommendation applies, and the intervention, that is, the characterization of what should be performed or not performed on the given population [11]. QIs are commonly derived from evidence-based clinical practice guidelines, so their content can be represented using the same structure. Therefore, in the first step of developing computer-interpretable representations, we decomposed all 10 DIVI QIs and their 31 separately measurable subindicators into the PICO components population and intervention. To formally define the contents of each component, we further decomposed them into separate medical concepts connected using logical operators (eg, “AND” and “OR”).

This decomposition process requires a significant amount of medical domain knowledge, as QIs are defined by intensive care clinicians for other intensive care clinicians and thus often omit details obvious to these domain specialists. To verify that the interpretations made during the first step of the translation into computer-interpretable representations align with the intent of the expert clinicians who defined the QIs, we concluded the logical formalization with a comprehensive expert review. This review involved a group of 9 expert clinicians from 6 different university hospitals, including the primary authors of the QIs in intensive care medicine for Germany and representatives of the Quality and Economy Section of the DIVI, as well as 4 medical data specialists, including representatives of the Information Technology Section of DIVI. The review’s goal was to validate that the decomposition of the QIs was performed accurately and to identify any required specifications beyond the level of detail provided by the published narrative version of the QIs. All required changes defined in the expert clinician review were iteratively fed back to the team performing the decomposition until no further changes were required.

To facilitate error-free communication between technical specialists and clinicians in the review process, we defined a format to represent the content of QIs that is human-readable, but structured and unambiguous. This “intermediate representation” can be easily understood by both groups, as it is free of any implicit clinical knowledge that technical specialists might not have, and it can also be understood without any deeper technical understanding that clinicians might not have. As the intermediate representation, we used sentences composed only of medical concepts (eg, “intensive care patient”), logical connectors (eg, AND, OR, and NOT), and numerical quantifiers (eg, “=1,” “>3,” and “≤5”).

### Semantic Annotation of the QIs

To ensure semantic interoperability, the decomposed QIs were semantically annotated using international standard vocabularies. We mapped each medical concept included in any logical term of the decomposed QIs to concepts from the terminologies Logical Observation Identifiers Names and Codes (LOINC) and Systemized Nomenclature of Medicine—Clinical Terms (SNOMED CT; international version, US version, and UK version). LOINC was used for laboratory tests and medical device data outputs, and SNOMED CT was used for all other domains. For cases where no matching concept could be found in either LOINC or SNOMED CT, we defined appropriate concepts in a supplementary code system.

### Development of the FHIR Instances

We represented the decomposed and semantically mapped QIs as FHIR instances according to the Clinical Practice Guidelines (CPG) on Evidence-Based Medicine (EBM) on the FHIR implementation guide (version 1.2.0).

Since structural indicators, unlike process and outcome indicators, typically do not rely on clinical data but instead on information often unavailable electronically (eg, physical and equipment resources) or only available from specialized databases (eg, staffing levels), and since they generally remain stable over long periods, they are usually evaluated at specific intervals rather than continuously. Given that the effort to make all necessary data electronically accessible for automated evaluation exceeds that of manual evaluation for these periodic assessments, the benefit of automating structure indicator evaluation is relatively low. Consequently, structural indicators were excluded from implementation in CPG-on-EBMonFHIR in this project.

The FHIR instances were created using FHIR Shorthand (FSH; version 3.0.0; HL7). The FSH code was converted via the SUSHI converter (version 3.14.0; HL7), a tool for compiling FSH into standard FHIR represented in JSON format. Technical validation against the CPG-on-EBMonFHIR profiles was conducted using the HL7 FHIR Validator (version 6.5.15) implemented in the Python package FSH Validator (version 0.3.5; [12]). Development was performed with code versioning on GitHub.

### Back-Translation of FHIR Instances for Concluding Clinical Review

As a final validation step, a data specialist who was unaware of the original narrative definitions of the QIs translated the FHIR-encoded QIs into the earlier-described intermediate representation format that could be understood and reviewed by our panel of clinicians. By this “back-translation,” we ensure that the FHIR-encoded structured representations truly and accurately reflect the intent of the original narrative definitions.

In detail, the back-translation was performed as follows: for each population-intervention pair of a single QI—represented as instances of the RecommendationPlan profile—the individual population characteristics as defined in the RecommendationEligibilityCriteria instances and the individual intervention characteristics as defined in the RecommendationAction instances were transformed into a narrative form using the display names of the associated medical concepts, connected using Boolean operators (AND, OR, and NOT).

These narrative representations were then subject to joint review sessions among a group of 9 expert clinicians from 6 different university hospitals, including the authors of the original QIs, the spokesperson and deputy spokesperson of the Quality and Economics Section, and the spokesperson of the Information Technology Section of the DIVI. While no formal checklist or rubric was applied during the review, clinicians evaluated whether the back-translated representations fully captured the clinical intent and specificity of the original QIs. Acceptable representations were those that matched the intended population and intervention logic. Discrepancies, such as overspecified, underspecified, or misinterpreted elements, were discussed in detail and revised until consensus was reached among the clinical reviewers.

Following the correction of the deviations identified in these review sessions, along with minor adjustments based on change requests to the QIs that the clinical expert group became aware of during this review, the here-developed computer-interpretable QIs were approved for release by the spokespersons of the Quality and Economics Section and the Information Technology Section of DIVI as the official digital representations of the DIVI QIs for intensive care medicine.

The release of the FHIR instances and postrelease maintenance are managed on GitHub using GitHub Issues and following a previously published workflow [13].

### Ethical Considerations

This study did not involve any experiments or the use of clinical data; therefore, ethics approval was not required. After consultation with our local ethics committee, no ethics exemption letter was obtained, as the ethics approval process is not applicable to the purely technical nature of this work.

## Results

### Logical Formalization of the Contents of the QIs

Table 1 provides an overview of the 10 QIs, detailing the population and intervention components for each associated indicator and its classification as process, structure, or outcome. Each QI is decomposed into distinct medical concepts that characterize the population and intervention, along with their logical relationships.

**Table 1:** Overview of QIs^a,b^.

QI, title and number		Type	Population	Intervention
**QI1: daily multiprofessional and interdisciplinary visit with documentation of daily goals**				
	1a	Process	Intensive care patient	Daily multiprofessional visit with participation of an intensive care specialist
	1b	Process	Intensive care patient	Daily definition of daily goals
**QI2: management of sedation, analgesia, and delirium**				
	2a	Process	Intensive care patient	1× per shift: measurement sedation score AND measurement pain score AND measurement delirium score
	2b	Outcome	Intensive care patient	Adequate sedation (RASS^c^ –1 to +1) AND pain freedom (NRS^d^≤3 OR VAS^e^≤3 OR BPS^f^≤3) AND no delirium (CAM-ICU^g^=0 AND ICSDC^h^=0)
	2c	Structure	N/A^i^	SOP^j^ for sedation available AND SOP for analgesia available AND SOP for delirium available
**QI3: patient-adapted ventilation (for severe lung failure)**				
	3a	Process	Intensive care patient AND severe lung failure AND invasive ventilation	Limitation of tidal volume≤6-7 mL/kg
	3b	Process	Intensive care patient AND severe lung failure AND invasive ventilation	Limitation of plateau pressure≤30 cm H_2_O
	3c	Process	Intensive care patient AND severe lung failure AND invasive ventilation	Limitation of driving pressure≤15 cm H_2_O
	3d	Process	Intensive care patient AND severe lung failure AND invasive ventilation	Individualized PEEP^k^ setting (according to ARDS^l^net-PEEP table)
	3e	Structure	N/A	SOP for ventilation available
**QI4: early weaning from invasive ventilation**				
	4a	Process	Intensive care patient AND invasive ventilation AND no ventilation at home before admission	Daily: weaning ability evaluated OR weaning attempt documented
	4b	Outcome	Intensive care patient AND invasive ventilation AND no ventilation at home before admission	No transfer with ventilation at home
	4c	Structure	N/A	SOP for weaning available
**QI5: monitoring of infection prevention measures**				
	5a	Process	Intensive care patient AND invasive device	Daily documentation of indication
	5b	Process	Intensive care patient	Consumption of hand disinfectant >80-100 L per 1000 days
	5c	Outcome	Intensive care patient AND invasive device	Low number of device infections/number of devices
	5d	Outcome	Intensive care patient AND invasive ventilation	Low number of ventilator-associated pneumonia
	5e	Structure	N/A	SOP for infection prevention available
	5f	Structure	N/A	Participation in ITS-KISS of NRZ^m^ or alternative structural benchmark
**QI6: measures for infection management**				
	6a	Process	Intensive care patient AND antibiotics prescription	For each prescription: documentation of indication AND documentation of focus AND documentation of duration of therapy
	6b	Outcome	Intensive care patient	Blood cultures ≥80 cultures per 1000 days
	6c	Structure	N/A	SOP for infection management available
**QI7: patient-adapted clinical nutrition**				
	7a	Process	Intensive care patient AND expected insufficient nutrition AND no contraindication for enteral nutrition	Clinical nutrition within 24 hours of admission
	7b	Process	Intensive care patient AND clinically nourished AND BMI≤30 kg/m^2^	Calorie intake≥calorie goal
	7c	Structure	N/A	SOP for nutrition therapy available
**QI8: structured communication with patients and family members**				
	8a	Process	Intensive care patient AND length of stay>72 hours	Conversation with specialist or consultant within 72 hours after admission
	8b	Process	Intensive care patient AND length of stay>72 hours	Conversation with specialist or consultant
**QI9: early mobilization**				
	9a	Process	Intensive care patient AND no nonmobilization order	Mobilization within 24 hours after admission
	9b	Process	Intensive care patient AND no nonmobilization order	Daily mobilization
	9c	Structure	N/A	SOP for early mobilization available
**QI10: intensive care unit management**				
	10a	Structure	N/A	Management of the ward by a specialist with the additional qualification of intensive care medicine who has no other clinical duties Medical presence during core working hours: >6 months experience up to specialist depending on hospital level Medical presence over 24 hours: >3 months experience Nursing ward manager with further specialist training AND studies or further management training Nursing staff ratio according to INPULS^n^ system depending on the complexity of treatment 1:1 to 1:3 (nursing staff:patients) Proportion of nursing professionals ≥30%

^a^QI: quality indicator.

^b^Criteria that need to be simultaneously fulfilled are connected using “AND”; criteria where one or the other should be fulfilled are connected using “OR.”

^c^RASS: Richmond Agitation-Sedation Scale.

^d^NRS: Numeric Rating Scale.

^e^VAS: Visual Analog Scale.

^f^BPS: Behavioral Pain Scale.

^g^CAM-ICU: Confusion Assessment Method for Intensive Care Unit.

^h^ICSDC: Intensive Care Delirium Screening Checklist.

^i^N/A: not applicable.

^j^SOP: standard operating procedure.

^k^PEEP: positive end-expiratory pressure.

^l^ARDS: acute respiratory distress syndrome.

^m^ITS-KISS of NRZ: Intensive Care Hospital Infection Surveillance System by the National Reference Center for Surveillance of Nosocomial Infections.

^n^INPULS: Intensivpflege und Leistungserfassungssystem (intensive care and performance recording system).

### Semantic Annotation of the QIs

Table 2 presents the semantic mappings for each QI, listing all medical concepts used in the indicators and their mappings to the standardized vocabularies LOINC and SNOMED. When existing vocabularies did not include an appropriate representation of a medical concept, we defined custom codes in the DIVI QI Semantic Terms (DIVI-QI-S) code system.

**Table 2:** Overview of semantic concepts.

Concept				OMOP^a^-ID		Vocabulary		Code
**QI1^b^**								
	**Intensive care patient**							
		Episode of care	46236992		LOINC^c^		78030-4	
		Intensive care unit	4148981		SNOMED^d^		309904001	
		Care of intensive care unit patient	4046295		SNOMED		133903000	
	**Interprofessional visit**							
		Participation in multidisciplinary ward round	37151470		SNOMED		1236923003	
	**Specialist with additional qualification in intensive care medicine**							
		Attending	1177391		LOINC		LP269965-2	
		Physician with intensive care specialist certification	—^e^		DIVI-QI-S^f^		FA-ZB-ITS	
	**Definition of daily goals**							
		Setting health objective	44807965		SNOMED		838411000000104	
		Setting daily care objective	—		DIVI-QI-S		TAGESZIEL	
**QI2**								
	**Recording sedation level**							
		Assessment of sedation level	44808934		SNOMED		851211000000105	
	**Recording pain level**							
		Assessment of pain control	4153048		SNOMED		370778008	
	**Recording delirium status**							
		Assessment of delirium	37116854		SNOMED		733870009	
	**RASS^g^**							
		Richmond Agitation-Sedation Scale	36684829		SNOMED		4574410001244102	
	**NRS^h^**							
		Numeric Pain Rating Scale	37151627		SNOMED		1284852002	
	**VAS^i^**							
		Visual analog pain scale	4165600		SNOMED		273904000	
	**BPS^j^**							
		Behavioral Pain Scale	—		DIVI-QI-S		BPS	
	**CAM-ICU^k^**							
		Short confusion assessment method	44807161		SNOMED		824471000000102	
		Confusion assessment method for intensive care	—		DIVI-QI-S		CAM-ICU	
	**ICDSC^l^**							
		Intensive care delirium screening checklist	—		DIVI-QI-S		ICDSC	
**QI3**								
	**Respiratory failure (ARDS^m^)**							
		Acute respiratory distress syndrome	4195694		SNOMED		67782005	
	**Oxygenation index (P/F ratio)**							
		Horovitz index in arterial blood	3029943		LOINC		50984-4	
		Oxygenation index measurement	4193842		SNOMED		313558004	
		Oxygenation index	37393330		SNOMED		1015621000000108	
	**Invasive ventilation**							
		Invasive ventilation	44790095		SNOMED		226471000000101	
	**Tidal volume**							
		Tidal volume/ideal body weight (ARDSnet)	—		DIVI-QI-S		tvpibw	
	**Plateau pressure**							
		Plateau pressure	4139635		SNOMED		264907004	
		Pressure.max respiratory system airway—on ventilator	36306157		LOINC		76531-3	
		Peak inspiratory pressure	27913002		SNOMED		27913002	
	**Driving pressure**							
		Airway pressure delta—on ventilator	42527138		LOINC		76154-4	
	**PEEP^n^**							
		PEEP respiratory system—on ventilator	21490855		LOINC		76248-4	
		Positive end expiratory pressure	4353713		SNOMED		250854009	
**QI4**								
	**Evaluation of weaning ability**							
		Weaning from mechanically assisted ventilation	4072633		SNOMED		243174005	
		Evaluation procedure	4297090		SNOMED		386053000	
	**Weaning attempt**							
		Weaning from mechanically assisted ventilation commenced	37154097		SNOMED		1259865002	
	**Home ventilation**							
		Dependence on home ventilator	46273524		SNOMED		60631000119109	
**QI5**								
	**Invasive device=catheter**							
		Catheter	4060422		SNOMED		19923001	
	**Catheter indication**							
		Indication of	4044935		SNOMED		230165009	
		Central venous catheter	4179206		SNOMED		52124006	
	**Hand disinfectant consumption**							
		Disinfectant	4210570		SNOMED		311942001	
		Use	4080150		SNOMED		277889008	
	**Catheter infection**							
		CLABSI—central line–associated bloodstream infection	42537043		SNOMED		736152001	
	**Ventilator-associated pneumonia**							
		Ventilator-associated pneumonia	259992		SNOMED		429271009	
**QI6**								
	**Antibiotics prescription**							
		Antibiotic therapy	4085730		SNOMED		281789004	
	**Antibiotic indication**							
		Indication of	4044935		SNOMED		230165009	
		Antibiotic	40584020		SNOMED		41000005	
	**Antibiotics focus**							
		Infectious disease	432250		SNOMED		40733004	
		Site of	4155553		SNOMED		272737002	
	**Duration of antibiotic therapy**							
		Duration of therapy	4129945		SNOMED		261773006	
	**Blood culture**							
		Blood culture	4107893		SNOMED		30088009	
**QI7**								
	**Presumably inadequate nutrition**							
		Predicted inadequate energy intake	763398		SNOMED		440331000124103	
	**Clinical nutrition**							
		Feeding patient	4327347		SNOMED		75118006	
	**Contraindications for enteral nutrition**							
		Contraindication to	4011945		SNOMED		103306004	
		Enteral feeding	4042005		SNOMED		229912004	
	**BMI**							
		Body mass index	4245997		SNOMED		60621009	
	**Calorie intake**							
		Energy intake (synonym calorie intake)	37206982		SNOMED		787787004	
	**Individual calorie target**							
		Energy requirement (synonym recommended energy intake)	4022415		SNOMED		226244007	
**QI8**								
	**Admission to intensive care unit**							
		Admission to intensive care unit	4138933		SNOMED		305351004	
	**Patient or family interview**							
		Client participation	4022119		SNOMED		225330006	
**QI9**								
	**Mobilization**							
		Mobilization	4327195		SNOMED		74923002	
	**Ban on mobilization**							
		Recommendation to rest in bed	4079772		SNOMED		183074009	

^a^OMOP: Observational Medical Outcomes Partnership.

^b^QI: quality indicator.

^c^LOINC: Logical Observation Identifiers Names and Codes.

^d^SNOMED CT: Systemized Nomenclature of Medicine.

^e^DIVI-QI-S: DIVI QI Semantic Terms.

^f^Not available.

^g^RASS: Richmond Agitation-Sedation Scale.

^h^NRS: Numeric Rating Scale.

^i^VAS: Visual Analog Scale.

^j^BPS: Behavioral Pain Scale.

^k^CAM-ICU: Confusion Assessment Method for Intensive Care Unit.

^l^ICSDC: Intensive Care Delirium Screening Checklist.

^m^ARDS: acute respiratory distress syndrome.

^n^PEEP: positive end-expiratory pressure.

### Development of FHIR Instances

**Figure 2:**
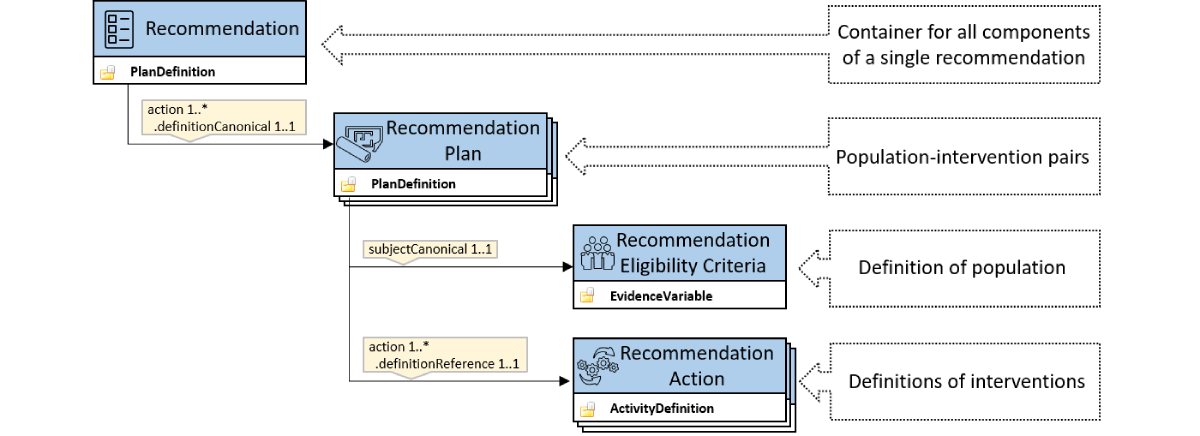
Resource structure and relationships for a single recommendation.

Each QI was represented by a single instance of a PlanDefinition resource using the Recommendation profile. As a single QI may define multiple different population-intervention pairs, each of these was described in PlanDefinition resources using the RecommendationPlan profile. Populations contained within the distinct population-intervention pairs were represented by EvidenceVariable resources using the RecommendationEligibilityCriteria profile, and interventions were represented by ActivityDefinition resources using the RecommendationAction profile (Figure 2).

**Figure 3:**
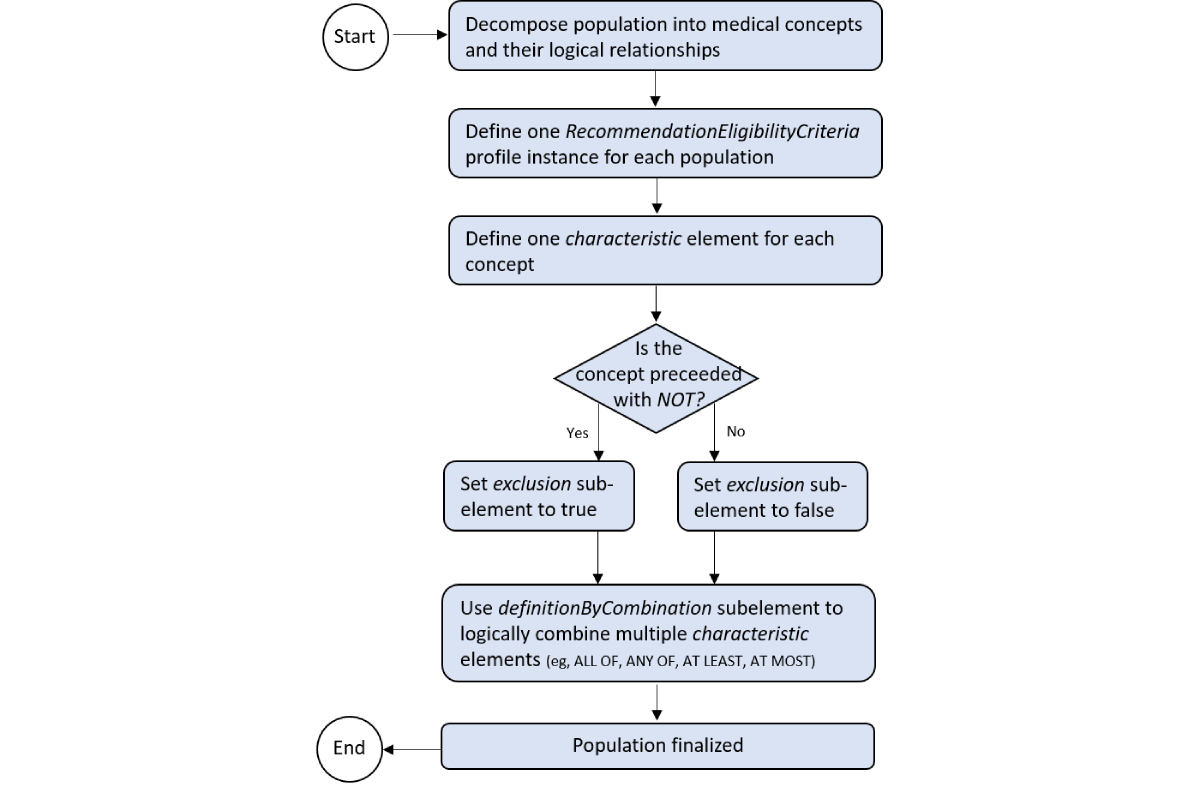
Procedural algorithm to define the populations in the RecommendationEligibilityCriteria profile.

Defining the populations from the decomposed and semantically annotated QIs was possible without limitation by representing each relevant concept using characteristic elements, which allowed the attachment of the concepts from the respective vocabularies in their definitions. Representing the logical relationships (eg, NOT, AND, and OR) between the separate characteristics within a population was realized using each characteristic’s exclusion subelement and definitionByCombination subelement. The procedural steps for defining populations in the RecommendationEligibilityCriteria profile are illustrated in Figure 3.

**Figure 4:**
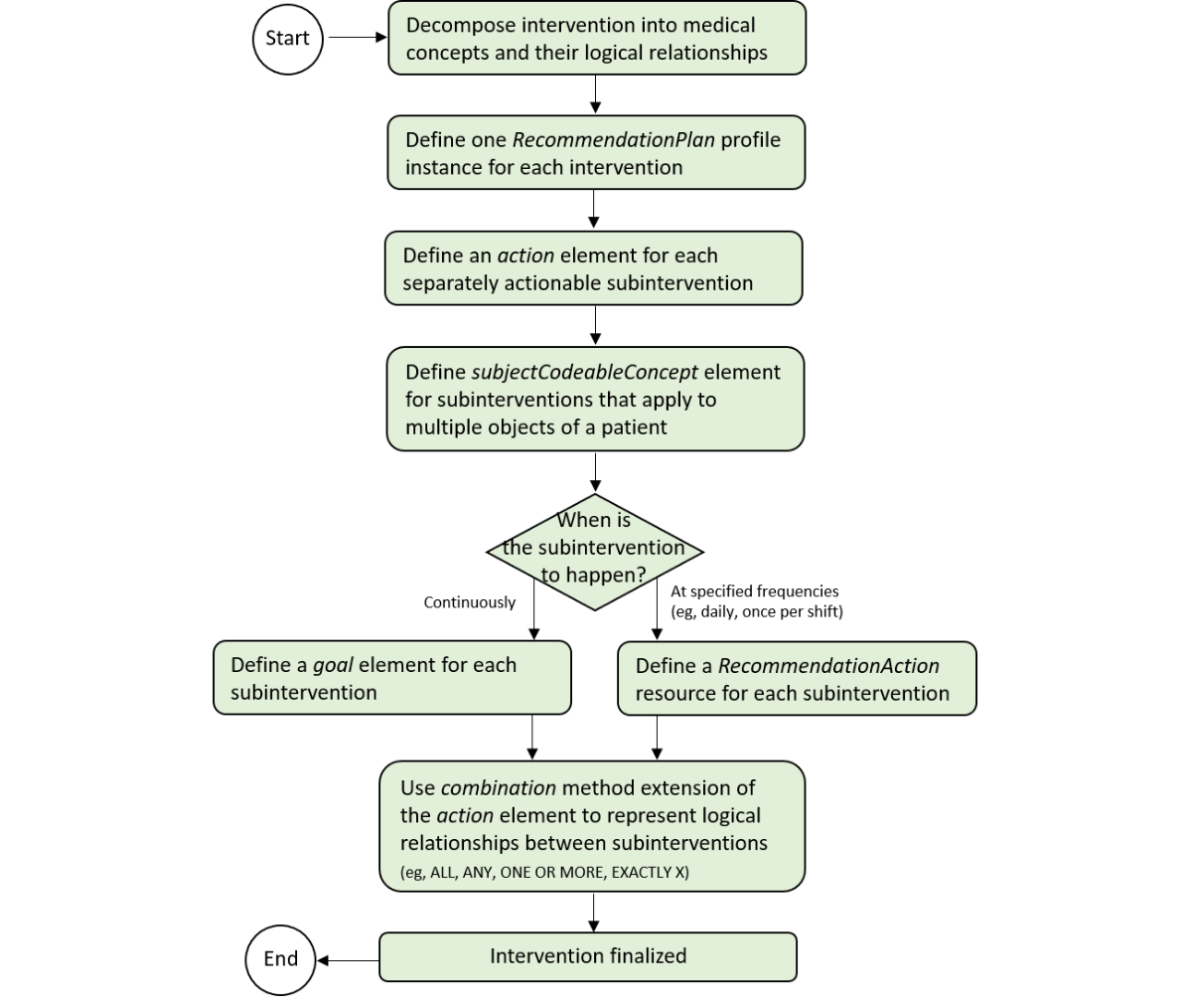
Procedural algorithm to define interventions in the RecommendationPlan profile.

Defining interventions from the decomposed and semantically annotated QIs required additional considerations compared to defining populations. Specifically, it was necessary to distinguish between interventions that are intended to occur (or not occur) at defined frequencies (eg, daily, once per shift, and 3 times per hour) and those that apply continuously and that focus on evaluating whether a value is in a specified range (eg, clinical score<X and technical device value between X and Y). Interventions intended to occur (or not occur) at defined frequencies can be represented appropriately as separate RecommendationAction instances, linked via action elements within the corresponding RecommendationPlan instance. In contrast, continuously applicable interventions are more appropriately represented as goal elements within the respective RecommendationPlan instance, which are also linked by the action elements within the same instance. For both types of interventions, the logical relationships between interventions can be specified using combination methods that represent a variety of logical relations that exceed simple AND or OR relations, including ALL, ANY, ONE OR MORE, EXACTLY X, and AT LEAST X. These distinctions and the procedural steps to define interventions in the RecommendationPlan profile are illustrated in Figure 4.

In terms of formal logic, nested Boolean conditions were handled using the definitionByCombination element of the EvidenceVariable resource (for populations) and the action element in the PlanDefinition resource (for interventions), allowing recursive nesting of logical expressions. Temporal conditions were represented using the FHIR Timing datatype. For example, QI2 specifies assessments “every 8 hours,” which was interpreted as once per clinical shift. This was modeled using 3 distinct ActivityDefinition instances (morning, afternoon, and night), each with a timing element specifying the relevant part of the day.

### Back-Translation of FHIR Instances for Concluding Clinical Review

Table 3 provides the back-translated narrative descriptions of each QI for the final clinical review. This back-translation process ensured that the computer-interpretable QIs accurately reflect the clinical intent of the original indicators. Discrepancies between the original narrative QIs and their computer-interpretable representations occur primarily when ambiguous statements from the original narrative QIs (eg, QI3: “Limitation of tidal volume≤6-7 mL/kg”) required further specification for an exact computer-interpretable representation (eg, QI3: “Tidal volume≤6 mL/kg ideal body weight”). We reviewed each translated instance with clinical experts, including the primary authors of the QIs, to verify consistency and clarity. The resulting level of granularity in the back-translated representations varies across indicators, reflecting the differing specificity and complexity of the original source material.

**Table 3:** Back-translation of QIs^a^.

Number		Population	Intervention	Specific information for examination
**QI1**				
	1a	Intensive care patients	Multiprofessional ward round carried out once a day with a specialist with additional qualifications	—^b^
	1b	Intensive care patients	Treatment goals set once a day	Treatment goal=daily therapy goal
**QI2**				
	2a	Intensive care patients	Depth of sedation assessed every 8 hours (more precisely: 6 AM-2 PM, 2 PM-10 PM, 10 PM-6 AM)	RASS^c^
	2a	Intensive care patients	Pain level assessed every 8 hours (more precisely: 6 AM-2 PM, 2 PM-10 PM, 10 PM-6 AM)	NRS^d^, VAS^e^, BPS^f^
	2a	Intensive care patients	Delirium status assessed every 8 hours (more precisely: 6 AM-2 PM, 2 PM-10 PM, 10 PM-6 AM)	CAM-ICU^g^, ICDSC^h^
	2b	Intensive care patients	Sedation depth within an acceptable range	RASS –1 to +1
	2b	Intensive care patients	Pain level within acceptable range	NRS, VAS, BPS=3
	2b	Intensive care patients	Delirium status in an acceptable range	CAM-ICU, ICDSC negative (=0)
**QI3**				
	3a	All intensive care patients with invasive ventilation and severe ARDS^i^ (oxygenation index<100)	Tidal volume≤6 mL/kg ideal body weight	Ideal body weight according to gender-adapted formulae from ARDSnet
	3b	All intensive care patients with invasive ventilation and severe ARDS (oxygenation index<100)	Ventilation plateau pressure≤30 cm H_2_O	—
	3c	All intensive care patients with invasive ventilation and severe ARDS (oxygenation index<100)	Driving pressure≤15 cm H_2_O	—
**QI4**				
	4a	All intensive care patients with invasive ventilation AND without pre-existing home ventilation	Ventilation weaning evaluated once a day or weaning trial carried out once a day	—
	4b	All intensive care patients with invasive ventilation AND without pre-existing home ventilation	No transfer to outpatient ventilation (nursing home, home)	—
**QI5**				
	5a	All intensive care patients with invasive devices	For each invasive device: the indication for retention is evaluated once a day	Invasive devices=all types of catheters that are considered device-associated infections
	5b	All intensive care patients with invasive devices	For every invasive device and no occurrence of device-associated infections	Device-associated infections, ventilator-associated infections
	5c	All intensive care patients	Consumption of hand disinfectant≥80 L per 1000 days	—
**QI6**				
	6a	All intensive care patients with antibiotics	For each antibiotic: the indication, focus, and duration of therapy are evaluated once a day	—
	6b	All intensive care patients with antibiotics	Number of blood cultures≥80 per 1000 days	—
**QI7**				
	7a	All intensive care patients with likely to have insufficient calorie intake AND no contraindication for enteral nutrition	Enteral nutrition started within 24 hours of admission	Expected low calorie intake=expected calorie intake<individual calorie requirement in the next 24 hours Can be easily integrated into the daily goals if “expected calorie intake is too low”
	7b	All intensive care patients without obesity and with clinical nutrition	Daily clinical nutrition with sufficient calorie intake	Obesity=BMI≥30 kg/m² Sufficient calorie intake=calorie intake≥individual calorie requirement
**QI8**				
	8a	All intensive care patients with a length of stay >72 hours	First patient or family interview conducted within the first 72 hours of admission	—
	8b	All intensive care patients with a length of stay >72 hours	Patient or relatives meeting held once a week	—
**QI9**				
	9a	All intensive care patients without an immobilization order	First mobilization carried out within the first 24 hours after admission	—
	9b	All intensive care patients without an immobilization order	Mobilization performed once a day	—

^a^QI: quality indicator.

^b^Not available.

^c^RASS: Richmond Agitation-Sedation Scale.

^d^NRS: Numeric Rating Scale.

^e^VAS: Visual Analog Scale.

^f^BPS: Behavioral Pain Scale.

^g^CAM-ICU: Confusion Assessment Method for Intensive Care Unit.

^h^ICSDC: Intensive Care Delirium Screening Checklist.

^i^ARDS: acute respiratory distress syndrome.

## Discussion

### Principal Findings

In this study, we developed computer-interpretable representations of the outcome and process QIs for intensive care medicine in Germany using the FHIR-based CPG-on-EBMonFHIR format. This included the structured specification of each indicator’s population (ie, the patients to whom it applies) and the intervention criteria (ie, intended actions), the semantical mapping of the individual criteria to international terminologies, the translation into FHIR, and their validation through iterative review with clinical experts. Our results demonstrate that this format allows a full representation of the DIVI QIs. Furthermore, we established a structured process to develop trustable computer-interpretable representations of QIs by combining stepwise formalization with repeated clinical expert validation, ensuring that the final representations align with clinical understanding and intent. To support consistency and reuse, we created procedural algorithms for encoding populations and interventions in FHIR.

These representations can be used in automated quality management systems to standardize quality assessments across health care facilities. Our structured process may serve as a blueprint for similar efforts in other specialties. The here-developed computer-interpretable QIs are openly available for reuse and ongoing maintenance. Future work will focus on piloting these indicators in real-world clinical systems and extending the framework to include structural indicators.

### Logical Formalization of the QIs and Back-Translation

The transformation of human-readable medical content into computer-interpretable representations is highly error-prone. One major complicating factor is that understanding the human-readable representation requires a high level of medical knowledge, while understanding the computer-interpretable representation requires a high level of technical knowledge. It is uncommon for one individual to have a high level of knowledge in both domains, so errors in the transformation process are difficult to detect. To overcome this challenge, we use an intermediate representation, which can be easily understood by both groups of specialists and thus facilitate error-free communication. This intermediate representation is used at the two handover points in the process, at which the content to be transformed is (1) handed from the clinicians to the technical specialists in the beginning and (2) back to the clinicians at the end. As the intermediate representation, we here used sentences composed only of medical concepts (eg, “intensive care patient”), logical connectors (eg, AND, OR, and NOT), and numerical quantifiers (eg, “=1,” “>3,” and “≤5”).

After decomposing the human-readable representations of the QIs into sentences in this intermediate representation, clinicians were able to review the result. As expected, the initial transformation contained a high number of errors, both in the logical structure and in the medical concepts. Therefore, we performed a repeated clinical review right at the first handover point at the beginning of the transformation process, as early detection of errors reduces wasteful effort and increases the overall efficiency of the process. The second clinical review is installed at the end of the transformation process to ensure that the final result of the computer-interpretable representations matches precisely with what was clinically intended. Again, the intermediate representation was used by performing a back-translation of the FHIR representations into the intermediate representation. The formalized nature of the intermediate representation, which closely matches the structure of the computer-interpretable representations, ensures a low rate of errors in this back-translation step. Thus, the clinician’s validation of the statements in the intermediate representation can be regarded as a high level of validity of computer-interpretable representations in the context of a highly specialized medical domain. In addition, even when the computer-interpretable representations are not used, the formulations in the intermediate language facilitate a highly comparable and standardized evaluation of the QIs. This is especially important for the evaluation of the effects associated with the measurement of QIs on patient outcomes [14]. As a limitation, although our expert review process involved multiple rounds of feedback from clinical experts, it did not use structured validation measures such as interrater agreement, predefined review checklists, or formal validation rubrics.

### Development of the FHIR Instances

Even though the CPG-on-EBMonFHIR implementation guide standardizes the content of FHIR to represent clinical guideline recommendations and QIs, considerable variability remains in how specific relations can be encoded. To reduce this variability and further harmonize the encoding of QIs in CPG-on-EBMonFHIR, we developed procedural algorithms that define how to encode populations and interventions in CPG-on-EBMonFHIR. The consequential reduction of variability in encoding not only facilitates the encoding process but also simplifies the development of software that integrates the resulting computer-interpretable QIs, as developers can rely on a consistent structure and infer meaning directly from it. In the development of the procedural algorithms to encode populations and interventions, we accounted for all aspects encountered in the QIs encoded during this project, ensuring complete coverage of their specific features. We further addressed more complex cases such as populations connected with a logical OR and interventions with logical relations like ALL, ANY, ONE OR MORE, EXACTLY X, and AT LEAST X. However, the coverage of all possible aspects is still far from complete, and further translations of QIs and guideline recommendations in CPG-on-EBMonFHIR will likely lead to further additions and refinement of the procedural algorithms. Once these algorithms cover all possible logical structures of QIs, they will allow for an automated transformation of content between the earlier-described intermediate language into CPG-on-EBMonFHIR and back, thus further reducing the risk of errors inherent in manual development of FHIR.

We did not produce FHIR-based representations for structural QIs (eg, room size, equipment availability, staffing levels, or standard operating procedures). Unlike process indicators, which vary across patients and time, structural indicators are relatively stable over time and do not differ between individual patients. Thus, structural and process indicators serve different purposes in clinical quality management. Process indicators require frequent monitoring at the individual patient level, often daily, whereas structural indicators are typically assessed at higher organizational levels, such as ward-level, and at less frequent intervals (eg, quarterly). Therefore, automating the monitoring of structural indicators provides limited benefit, as the effort of manual monitoring is already relatively low. Furthermore, automating structural indicators requires integrating data from various electronic systems (eg, rostering systems and facility management systems), unlike process indicators, which are all based on clinical data. While technically feasible, the substantial effort required for an automated monitoring of structural indicators typically outweighs the limited benefit achieved. Accordingly, from our clinician stakeholders’ perspective, there is no demand for FHIR-based representations of structural QIs. Even though structural indicators remain crucial for benchmarking across institutions, for this purpose, data can be more effectively collected through standardized data entry rather than through automated integration. As in such institutional benchmarking, the calculation of indicators based on the data entries is only implemented once in a central system, and the benefit of using computer-interpretable representations for this purpose is negligible. However, to ensure consistent evaluation across institutions, clear and unambiguous definitions are essential. Therefore, although we did not produce digitized FHIR representations for structural QIs, we explicitly defined all indicators, including structural ones, in our human-interpretable intermediate language to support broader harmonization and adoption (Table 1).

### Semantic Annotation

To ensure semantic interoperability, the decomposed QIs were semantically annotated using international standard vocabularies. We mapped each medical concept included in any logical term of a decomposed QI to a concept from LOINC or SNOMED CT. Even though these vocabularies are among the most comprehensive international standard vocabularies in medicine, their scope is still quite far from sufficient to cover all medical content. Of the 58 unique medical concepts identified in the QIs, 52 (90%) were successfully mapped to concepts in LOINC or SNOMED CT. The remaining 6 concepts, for which we defined entries in a supplementary vocabulary (DIVI-QI-S), were mostly specific to intensive care medicine. These included detailed descriptions of roles (“specialist with additional qualification in intensive care medicine”), measures (“setting daily care objective”), clinical scores (“Behavioral Pain Scale,” “Confusion Assessment Method for Intensive Care,” and “Intensive Care Delirium Screening Checklist”), or combined measures (“tidal volume/ideal body weight [ARDSnet]”). Expecting a similar lack of coverage for specific concepts can be found in all specific fields of medicine, and it would be useful to allow for a simpler and possibly distributed way of enlarging the scope of standard vocabularies. The DIVI-QI-S code system is publicly maintained via GitHub with issue-based change tracking. To support long-term interoperability, we plan to submit relevant concepts to SNOMED CT. Additionally, we are coordinating these efforts with the team developing the intensive care unit module of the German Medical Informatics Initiative core dataset, aiming to ensure consistency and alignment with national semantic standardization activities. While the use of nonstandard codes may limit out-of-the-box interoperability with systems relying strictly on international terminologies, this limitation is mitigated by providing openly maintained code definitions.

Next to the availability of semantic concepts, choosing the right level of complexity for a concept can also pose a challenge. The primary purpose of defining semantic concepts in computer-interpretable rules is to find the appropriate data items in the patient data for which the rules apply. Thus, ideally, rules would only contain semantic concepts that directly relate to specific data items in clinical databases. However, clinical databases are not standardized across health care facilities and differ in their data items. Therefore, it appears more appropriate to encode computer-interpretable rules with semantic concepts that best match the rules’ intended purpose rather than choosing semantic concepts that match items of a clinical database in some health care facilities, but are unavailable in others. For example, most of the QIs we encoded here apply to all “intensive care patients.” However, clinical databases typically do not contain a data item that holds whether a patient is an intensive care patient. This information must be inferred from other data items, typically from the current ward in which a patient is treated. In this case, the semantic concept “intensive care patient” is locally mapped to “patient treated in an intensive care unit.” However, using this data level specification directly in the computer-interpretable rule would limit its interoperability, as some health care facilities might have mixed wards with intensive care patients and nonintensive care patients or other organizational structures in which the type of patient cannot be inferred from the ward. In consequence, the highest interoperability can be achieved when semantic concepts in clinical rules are chosen to best match the intended purpose of the clinical rule, allowing every health care facility to map these concepts to those data items in the local database that best match the intended purpose.

### Generalizability to Other QI Sets

Although our 4-step workflow was here applied only to QIs from intensive care medicine—specifically, the German DIVI QIs—the components of the workflow (logical decomposition, semantic mapping, FHIR encoding with CPG-on-EBMonFHIR, and dual expert validation) are largely domain- and country-agnostic. Reusing this workflow to develop digital representations of QIs from other regions or medical specialties would mainly require (1) substituting local or specialty-specific terminologies during the semantic-mapping step and (2) convening an expert panel familiar with those indicators. In our case, the DIVI QIs required some disambiguation and refinement of clinical intent before they could be formally represented. A particular challenge was the inconsistent level of granularity across the original narrative QIs: some indicators were specified in much more detail than others. This issue will be addressed in the next version of the narrative indicators to enable more consistent digital implementation. This highlights the importance of the structured process, which allowed us to resolve ambiguities through iterative expert review. While the scalability of the approach to other specialties and regions remains to be shown, we expect that similar challenges such as ambiguous clinical intent or inconsistent granularity will arise elsewhere. Therefore, our structured workflow may offer a useful starting point for addressing these in other contexts.

### Summary and Clinical Implications

In this study, we developed trustable computer-interpretable representations of the QIs for intensive care medicine in Germany using the FHIR-based CPG-on-EBMonFHIR format. These can be used in automated evaluation software packages [15] to facilitate a standardized implementation and allow comparisons of QIs across health care facilities. Even when the computer-interpretable representations are not used, the formulations in the intermediate language allow for a highly comparable and standardized evaluation of the QIs.

Next to these primary results, we also designed a structured process to develop trustable computer-interpretable representations of clinical rules by stepwise formalization and repeated integration of clinical experts in the development process. This process is applied here for clinical QIs but could also be used to transform clinical guideline recommendations or other clinical rules into computer-interpretable representations, though further validation in other clinical domains will be necessary to confirm its general applicability. To further facilitate the transformation process, we developed procedural algorithms to define the specific resources required to represent clinical rules in CPG-on-EBMonFHIR. Future applications of these algorithms and their further refinement in the face of the requirements posed by clinical rules that differ in structure from those analyzed here will advance their development and allow for an automated transformation of clinical rules between the human-readable intermediate language and computer-interpretable FHIR.

While our work demonstrates that these QIs can be reliably represented and validated in a computer-interpretable format, real-world implementation within clinical information systems remains essential to assess their practical value. A logical next step is to integrate the FHIR-based representations into existing quality monitoring infrastructures, assess their feasibility, usability, and clinical acceptance, and define appropriate evaluation metrics to quantify their impact on quality management and patient outcomes.

## Appendix

Multimedia Appendix 1 STARE-HI checklist.

## Abbreviations

CPG: Clinical Practice Guidelines
DIVI: German Interdisciplinary Association of Critical Care and Emergency Medicine
DIVI-QI-S: DIVI QI Semantic Terms
EBM: Evidence-Based Medicine
FHIR: Fast Healthcare Interoperability Resources
FSH: FHIR Shorthand
HL7: Health Level Seven
LOINC: Logical Observation Identifiers Names and Codes
PICO: population, intervention, comparison, outcome
QI: quality indicator
SNOMED CT: Systemized Nomenclature of Medicine—Clinical Terms
STARE-HI: Statement on the Reporting of Evaluation Studies in Health Informatics

## Data Availability

The code of the computer-interpretable quality indicators developed in this project is available from the corresponding authors on reasonable request.
