# Supplementary material for: Computer-Interpretable Quality Indicators for Intensive Care Medicine: Development and Validation Study
Source: J Med Internet Res. 2025 Sep 26;27:e77077. doi: 10.2196/77077 (PMC12533511; doi:10.2196/77077)
Supplement: Multimedia Appendix 1 [file jmir_v27i1e77077_app1.pdf]

STARE-HI item	Item comprised in manuscript?
**Title**	☑ The title indicates the type of content evaluated and the study design.
**Abstract**	Abstract is structured and describes background, objectives, methods, results and conclusion
**Keywords**	Keywords are included
**Introduction**	
Scientific background	☑
Rationale for the study	☑
Objectives of the study	☑
**Study context**	
Organizational setting	☑
System details and system in use	n/a (no system evaluated)
**Methods**	
Study design	☑ Section “A structured process to develop trustable digital quality indicators”
Theoretical background	n/a (no theoretical background required)
Participants	☑ (Participating Clinician and Technical experts described)
Study flow	☑ Flow Diagram in Figure 1
Outcome measures or evaluation criteria	☑ Agreement of clinical experts with backtranslated quality indicators & technical validation
Methods for data acquisition and measurement	n/a
Methods for data analysis	n/a
**Results**	☑
Demographic and other study coverage data	☑ Quality Indicators listed in Table 1
Unexpected events during the study	n/a
Study findings and outcome data	☑ Table 1 and 2

STARE-HI item	Item comprised in manuscript?
Unexpected observations	n/a
**Discussion**	☑
Answers to study questions	☑ Beginning of Discussion
Strengths and weaknesses of the study	☑ Section “Summary and Clinical Implications”
Results in relation to other studies	n/a (first study on computer-interpretable quality indicators in intensive care medicine)
Meaning and generalizability of the study	☑ Sections “Generalizability to other Quality Indicator Sets” and “Summary and Clinical Implications”
Unanswered and new questions	☑ Sections “Generalizability to other Quality Indicator Sets” and “Summary and Clinical Implications”
**Conclusion**	☑ Section “Summary and Clinical Implications” in Discussion
**Authors’ contribution**	☑ Section “Author Contribution”
**Competing interests**	☑ Section “Conflicts of interest”
**Acknowledgement**	☑ Section “Acknowledgements”
**References**	☑ Section “References”
**Appendices**	☑ (this document)
